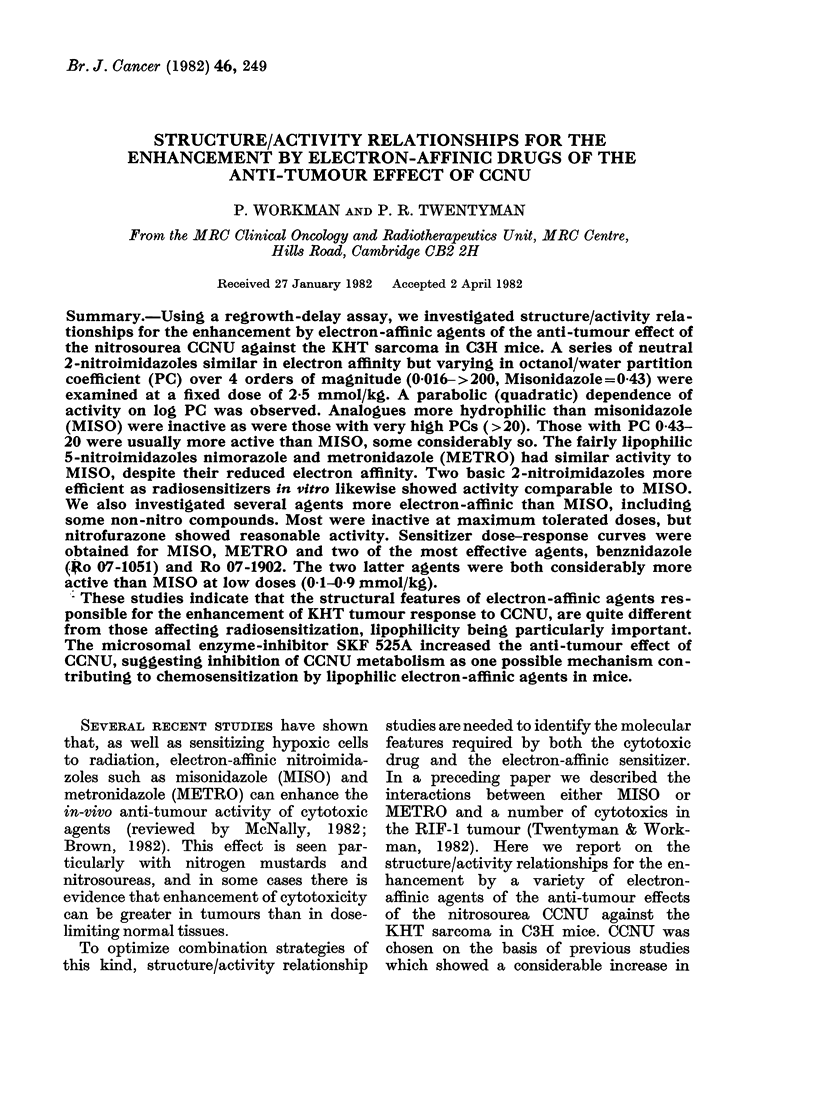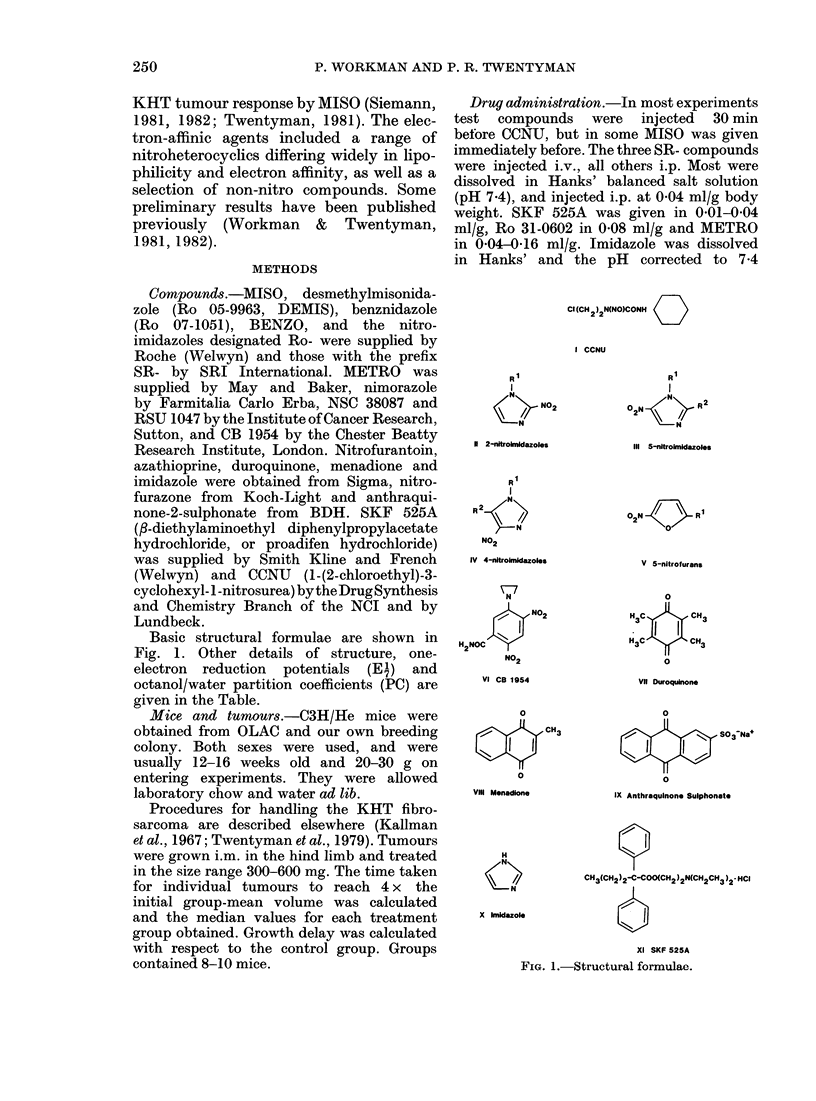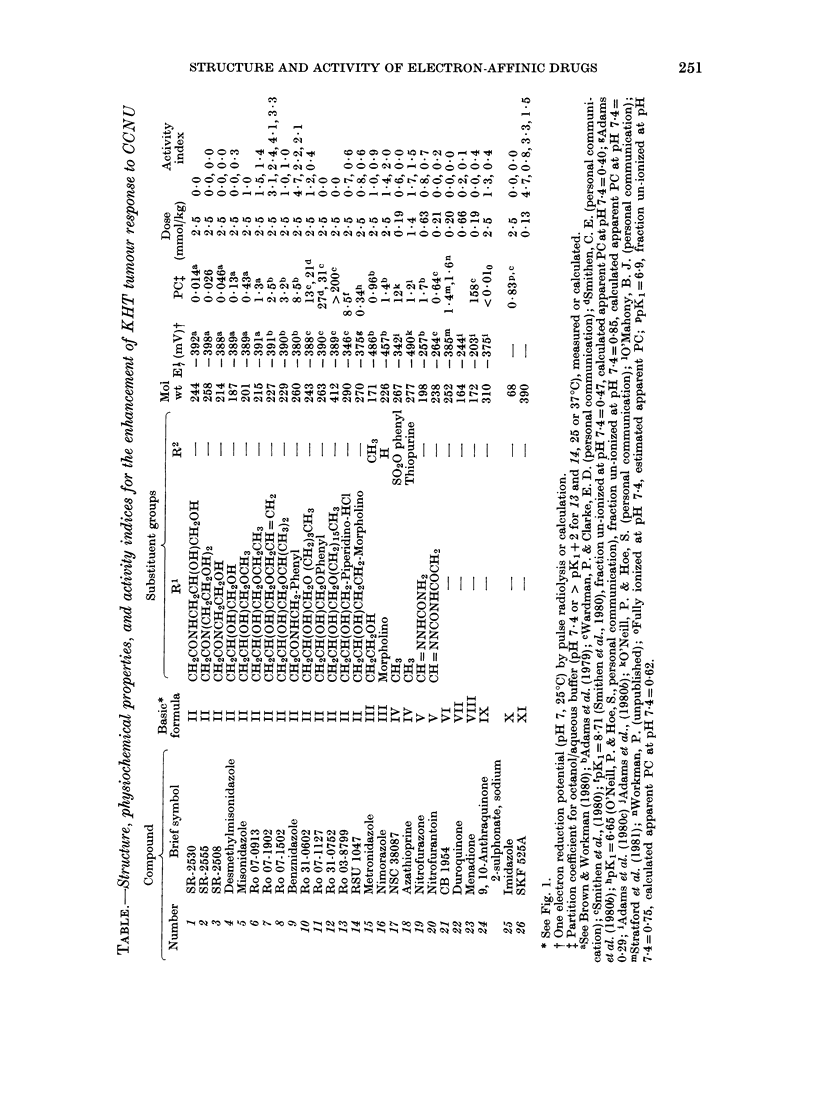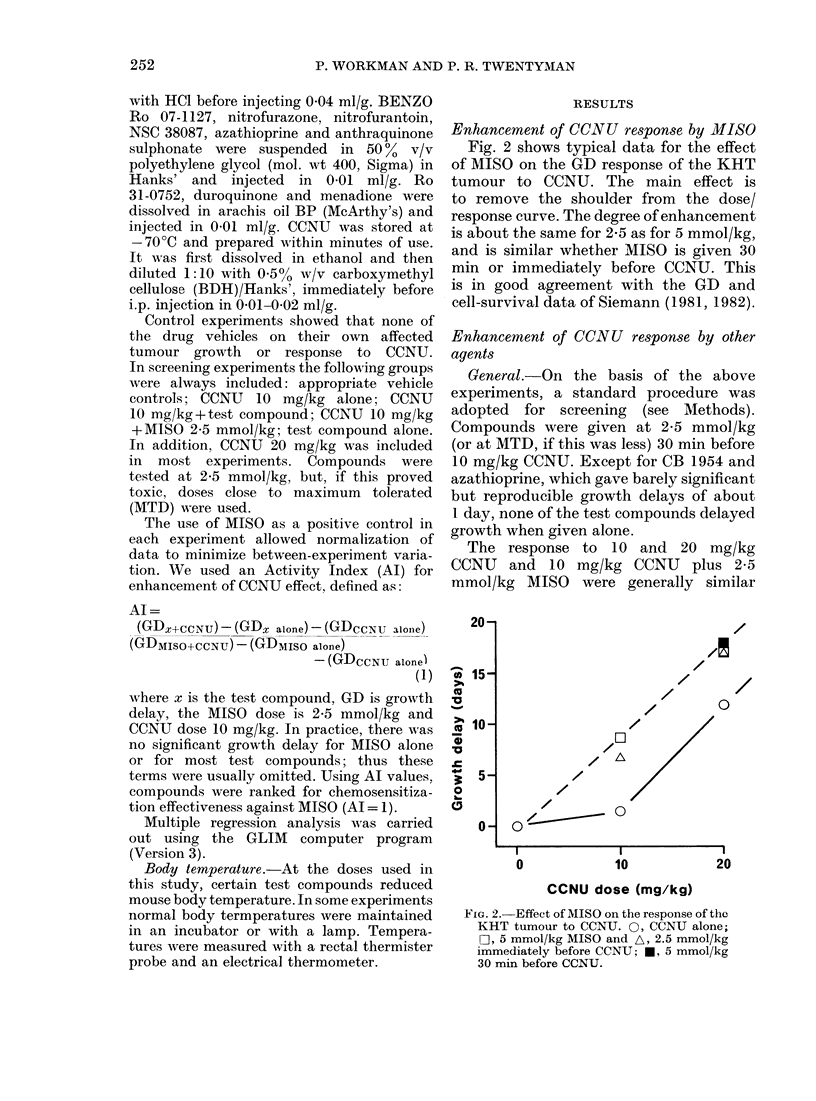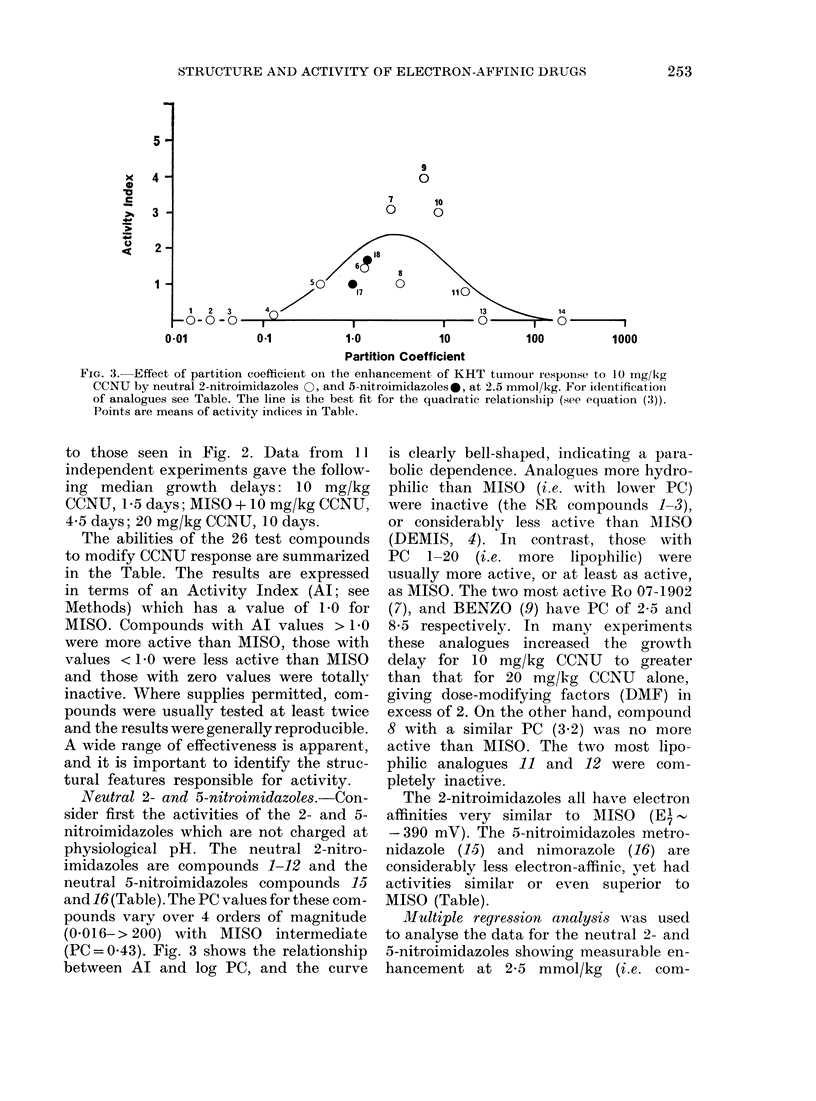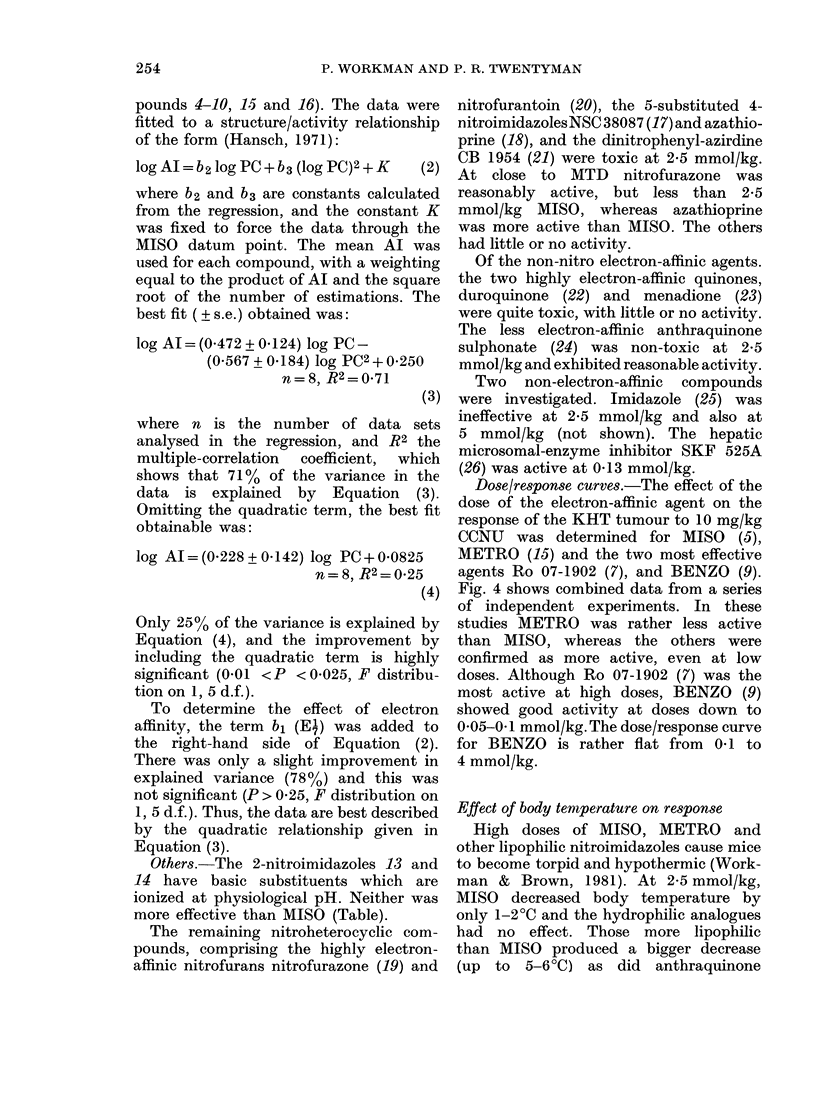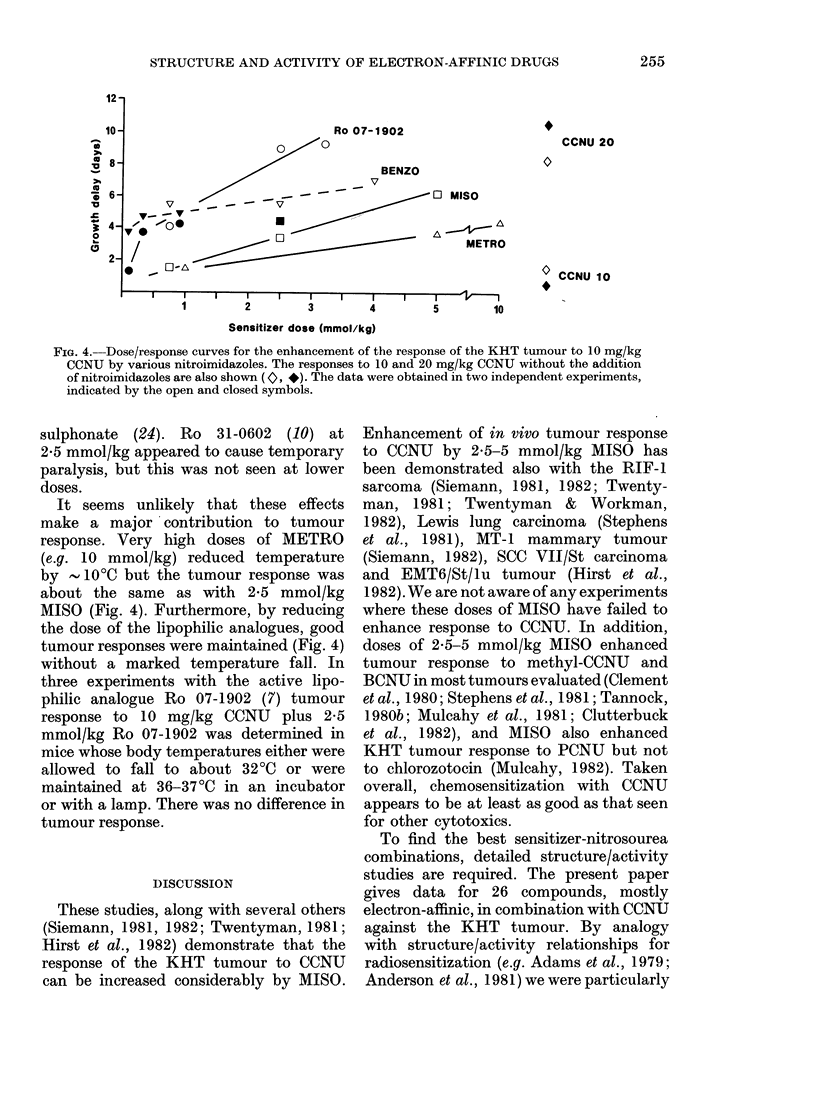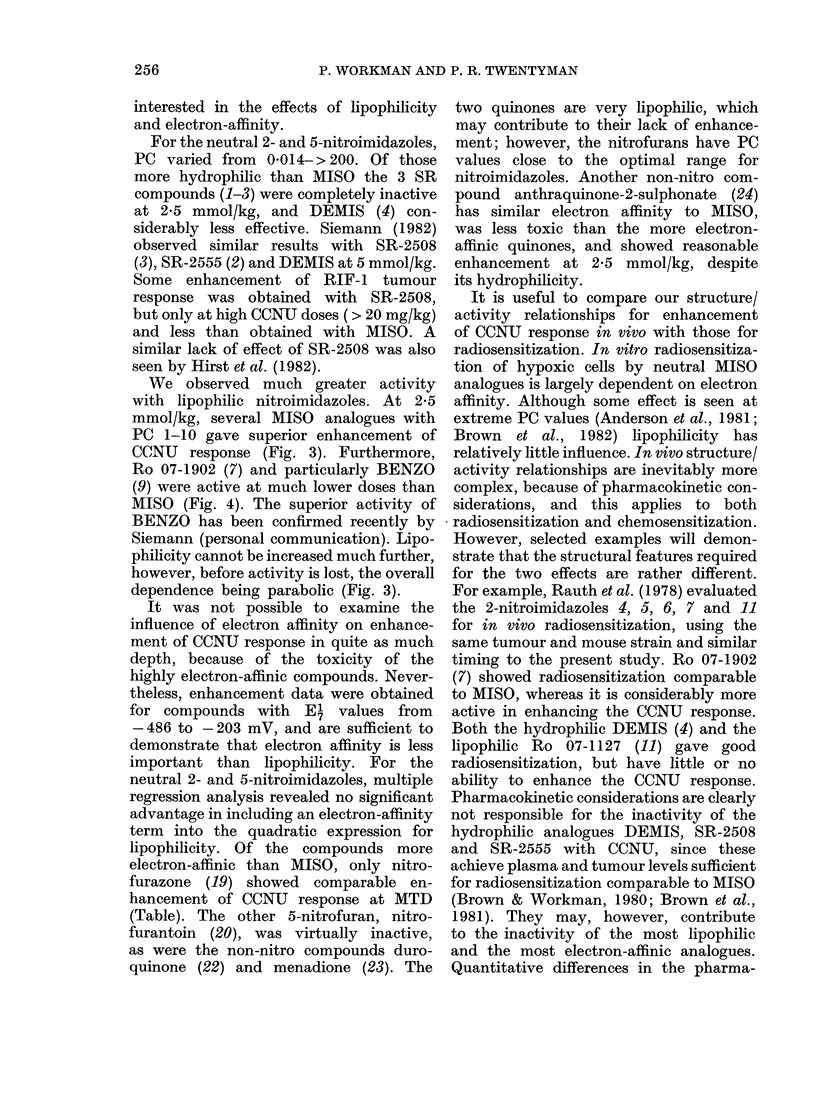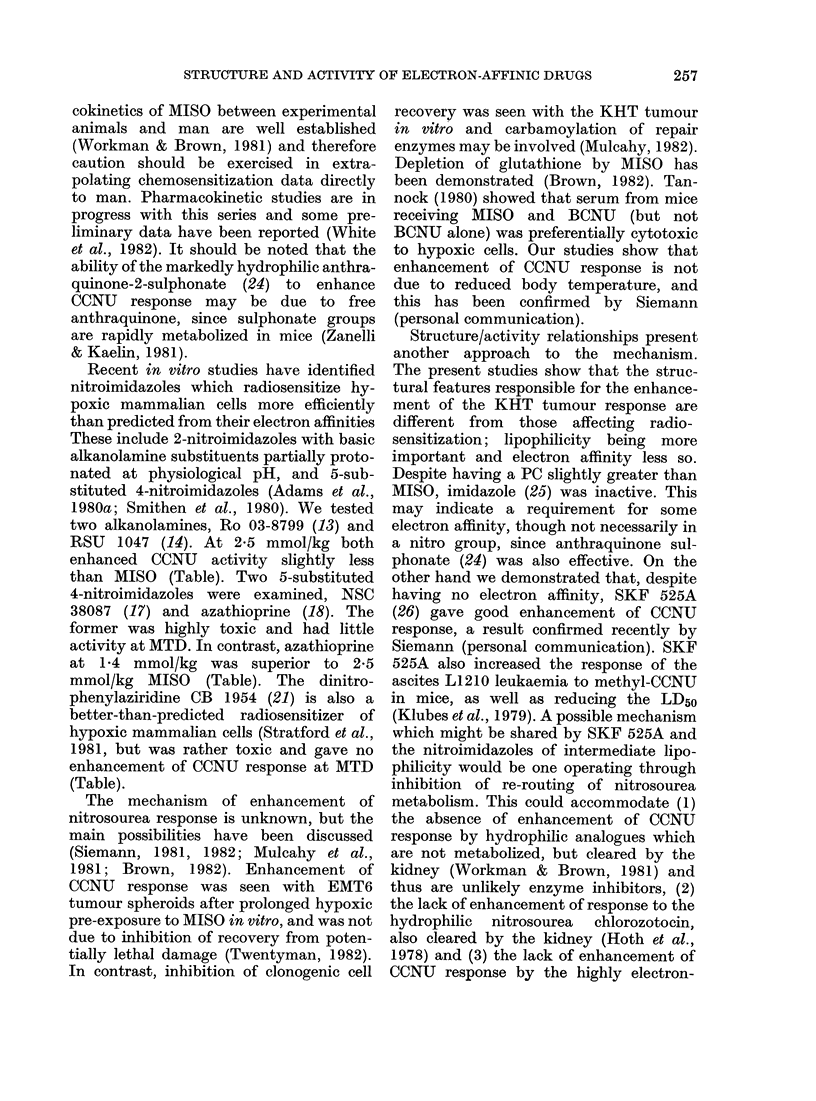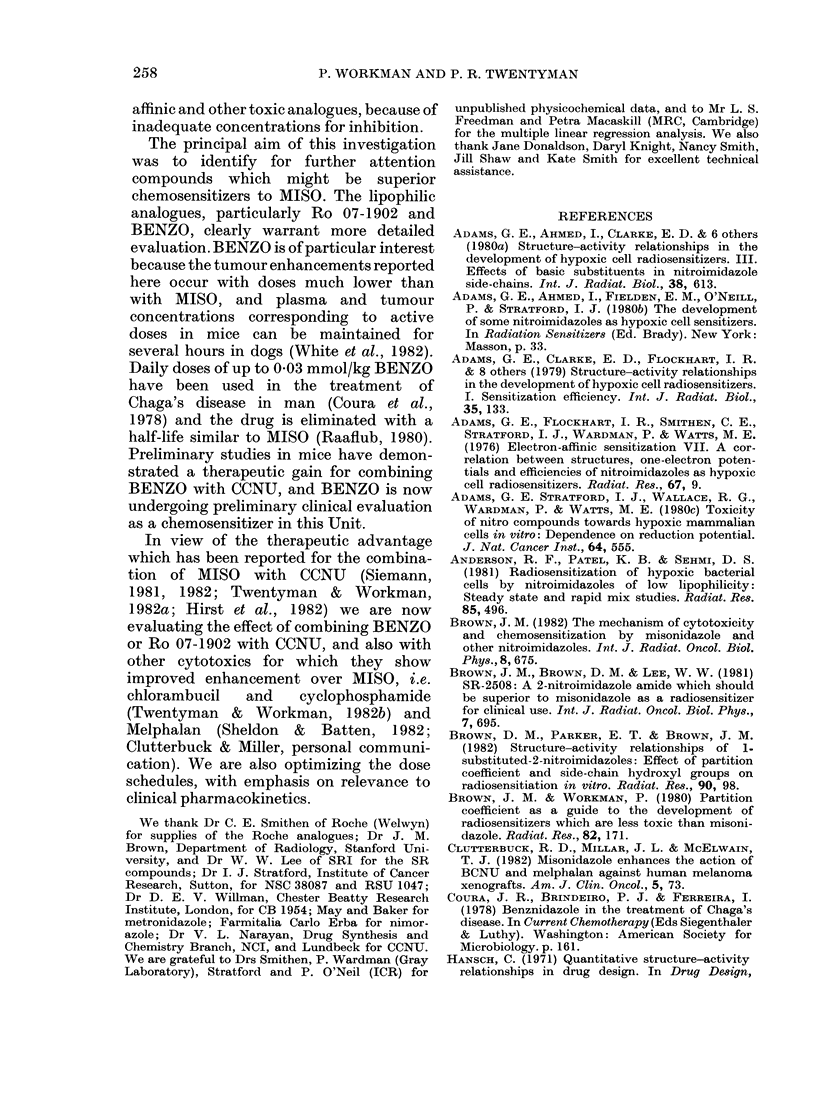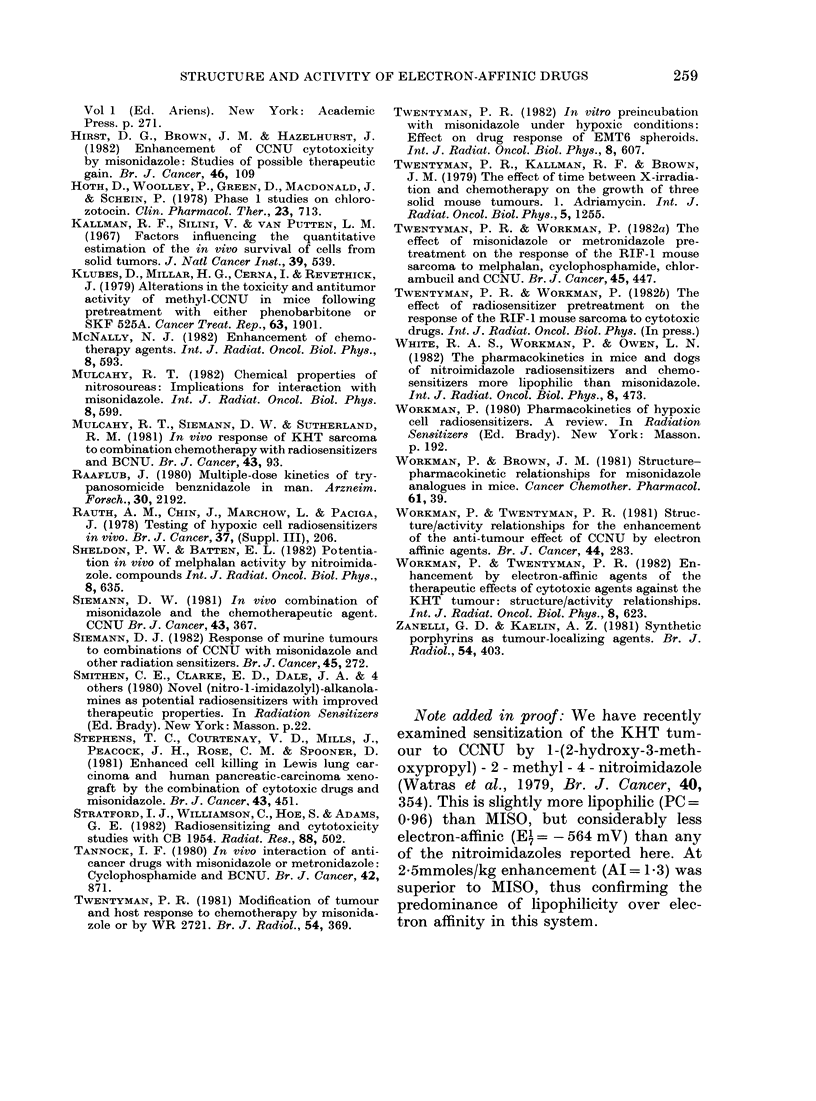# Structure/activity relationships for the enhancement by electron-affinic drugs of the anti-tumour effect of CCNU.

**DOI:** 10.1038/bjc.1982.190

**Published:** 1982-08

**Authors:** P. Workman, P. R. Twentyman

## Abstract

Using a regrowth-delay assay, we investigated structure/activity relationships for the enhancement by electron-affinic agents of the anti-tumour effect of the nitrosourea CCNU against the KHT sarcoma in C3H mice. A series of neutral 2-nitroimidazoles similar in electron affinity but varying in octanol/water partition coefficient (PC) over 4 orders of magnitude (0.016- greater than 200, Misonidazole = 0.43) were examined at a fixed dose of 2.5 mmol/kg. A parabolic (quadratic) dependence of activity on log PC was observed. Analogues more hydrophilic than misonidazole (MISO) were inactive as were those with very high PCs (greater than 20). Those with PC 0.43--20 were usually more active than MISO, some considerably so. The fairly lipophilic 5-nitroimidazoles nimorazole and metronidazole (METRO) had similar activity to MISO, despite their reduced electron affinity. Two basic 2-nitroimidazoles more efficient as radiosensitizers in vitro likewise showed activity comparable to MISO. We also investigated several agents more electron-affinic than MISO, including some non-nitro compounds. Most were inactive at maximum tolerated doses, but nitrofurazone showed reasonable activity. Sensitizer dose-response curves were obtained for MISO, METRO and two of the most effective agents, benznidazole (Ro 07-1051) and Ro 07-1902. The two latter agents were both considerably more active than MISO at low doses (0.1--0.9 mmol/kg). These studies indicate that the structural features of electron-affinic agents responsible for the enhancement of KHT tumour response to CCNU, are quite different from those affecting radiosensitization, lipophilicity being particularly important. The microsomal enzyme-inhibitor SKF 525A increased the anti-tumour effect of CCNU, suggesting inhibition of CCNU metabolism as one possible mechanism contributing to chemosensitization by lipophilic electron-affinic agents in mice.


*Br. J. Cancer* (1982) **46**, 249

### STRUCTURE/ACTIVITY RELATIONSHIPS FOR THE ENHANCEMENT BY ELECTRON-AFFINIC DRUGS OF THE ANTI-TUMOUR EFFECT OF CCNU

P. WORKMAN AND P. R. TWENTYMAN

*From the MRC Clinical Oncology and Radiotherapeutics Unit, MRC Centre, Hills Road, Cambridge CB2 2H*

Received 27 January 1982 Accepted 2 April 1982

**Summary.—Using a regrowth-delay assay, we investigated structure/activity relationships for the enhancement by electron-affinic agents of the anti-tumour effect of the nitrosourea CCNU against the KHT sarcoma in C3H mice. A series of neutral 2-nitroimidazoles similar in electron affinity but varying in octanol/water partition coefficient (PC) over 4 orders of magnitude (0·016–>200, Misonidazole=0·43) were examined at a fixed dose of 2·5 mmol/kg. A parabolic (quadratic) dependence of activity on log PC was observed. Analogues more hydrophilic than misonidazole (MISO) were inactive as were those with very high PCs (>20). Those with PC 0·43–20 were usually more active than MISO, some considerably so. The fairly lipophilic 5-nitroimidazoles nimorazole and metronidazole (METRO) had similar activity to MISO, despite their reduced electron affinity. Two basic 2-nitroimidazoles more efficient as radiosensitizers *in vitro* likewise showed activity comparable to MISO. We also investigated several agents more electron-affinic than MISO, including some non-nitro compounds. Most were inactive at maximum tolerated doses, but nitrofurazone showed reasonable activity. Sensitizer dose–response curves were obtained for MISO, METRO and two of the most effective agents, benznidazole (Ro 07-1051) and Ro 07-1902. The two latter agents were both considerably more active than MISO at low doses (0·1–0·9 mmol/kg).**

**These studies indicate that the structural features of electron-affinic agents responsible for the enhancement of KHT tumour response to CCNU, are quite different from those affecting radiosensitization, lipophilicity being particularly important. The microsomal enzyme-inhibitor SKF 525A increased the anti-tumour effect of CCNU, suggesting inhibition of CCNU metabolism as one possible mechanism contributing to chemosensitization by lipophilic electron-affinic agents in mice.**

SEVERAL RECENT STUDIES have shown that, as well as sensitizing hypoxic cells to radiation, electron-affinic nitroimidazoles such as misonidazole (MISO) and metronidazole (METRO) can enhance the *in-vivo* anti-tumour activity of cytotoxic agents (reviewed by McNally, 1982; Brown, 1982). This effect is seen particularly with nitrogen mustards and nitrosoureas, and in some cases there is evidence that enhancement of cytotoxicity can be greater in tumours than in dose-limiting normal tissues.

To optimize combination strategies of this kind, structure/activity relationship studies are needed to identify the molecular features required by both the cytotoxic drug and the electron-affinic sensitizer. In a preceding paper we described the interactions between either MISO or METRO and a number of cytotoxics in the RIF-1 tumour (Twentyman & Workman, 1982). Here we report on the structure/activity relationships for the enhancement by a variety of electron-affinic agents of the anti-tumour effects of the nitrosourea CCNU against the KHT sarcoma in C3H mice. CCNU was chosen on the basis of previous studies which showed a considerable increase in

KHT tumour response by MISO (Siemann, 1981, 1982; Twentyman, 1981). The electron-affinic agents included a range of nitroheterocyclics differing widely in lipophilicity and electron affinity, as well as a selection of non-nitro compounds. Some preliminary results have been published previously (Workman & Twentyman, 1981, 1982).

### METHODS

*Compounds.*—MISO, desmethylmisonidazole (Ro 05-9963, DEMIS), benznidazole (Ro 07-1051), BENZO, and the nitroimidazoles designated Ro- were supplied by Roche (Welwyn) and those with the prefix SR- by SRI International. METRO was supplied by May and Baker, nimorazole by Farmitalia Carlo Erba, NSC 38087 and RSU 1047 by the Institute of Cancer Research, Sutton, and CB 1954 by the Chester Beatty Research Institute, London. Nitrofurantoin, azathioprine, duroquinone, menadione and imidazole were obtained from Sigma, nitrofurazone from Koch-Light and anthraquinone-2-sulphonate from BDH. SKF 525A ($\beta$-diethylaminoethyl diphenylpropylacetate hydrochloride, or proadifen hydrochloride) was supplied by Smith Kline and French (Welwyn) and CCNU (1-(2-chloroethyl)-3-cyclohexyl-1-nitrosurea) by the Drug Synthesis and Chemistry Branch of the NCI and by Lundbeck.

Basic structural formulae are shown in Fig. 1. Other details of structure, one-electron reduction potentials ($E^{1}_{7}$) and octanol/water partition coefficients (PC) are given in the Table.

*Mice and tumours.*—C3H/He mice were obtained from OLAC and our own breeding colony. Both sexes were used, and were usually 12–16 weeks old and 20–30 g on entering experiments. They were allowed laboratory chow and water *ad lib.*

Procedures for handling the KHT fibrosarcoma are described elsewhere (Kallman *et al.*, 1967; Twentyman *et al.*, 1979). Tumours were grown i.m. in the hind limb and treated in the size range 300–600 mg. The time taken for individual tumours to reach 4× the initial group-mean volume was calculated and the median values for each treatment group obtained. Growth delay was calculated with respect to the control group. Groups contained 8–10 mice.

*Drug administration.*—In most experiments test compounds were injected 30 min before CCNU, but in some MISO was given immediately before. The three SR- compounds were injected i.v., all others i.p. Most were dissolved in Hanks' balanced salt solution (pH 7·4), and injected i.p. at 0·04 ml/g body weight. SKF 525A was given in 0·01–0·04 ml/g, Ro 31-0602 in 0·08 ml/g and METRO in 0·04–0·16 ml/g. Imidazole was dissolved in Hanks' and the pH corrected to 7·4

Fig. 1.—Structural formulae.

TABLE.—*Structure, physiochemical properties, and activity indices for the enhancement of KHT tumour response to CCNU*

| Compound | | Basic* | Substituent groups | | Mol | | | Dose | Activity |
|---|---|---|---|---|---|---|---|---|---|
| Number | Brief symbol | formula | $R^1$ | $R^2$ | wt | $E^1_7$ (mV)† | PC‡ | (mmol/kg) | index |
| *1* | SR-2530 | II | $CH_2CONHCH_2CH(OH)CH_2OH$ | — | 244 | −392[a] | 0·014[a] | 2·5 | 0·0 |
| *2* | SR-2555 | II | $CH_2CON(CH_2CH_2OH)_2$ | — | 258 | −398[a] | 0·026 | 2·5 | 0·0, 0·0 |
| *3* | SR-2508 | II | $CH_2CONCH_2CH_2OH$ | — | 214 | −388[a] | 0·046[a] | 2·5 | 0·0, 0·0 |
| *4* | Desmethylmisonidazole | II | $CH_2CH(OH)CH_2OH$ | — | 187 | −389[a] | 0·13[a] | 2·5 | 0·0, 0·3 |
| *5* | Misonidazole | II | $CH_2CH(OH)CH_2OCH_3$ | — | 201 | −389[a] | 0·43[a] | 2·5 | 1·0 |
| *6* | Ro 07-0913 | II | $CH_2CH(OH)CH_2OCH_2CH_3$ | — | 215 | −391[a] | 1·3[a] | 2·5 | 1·5, 1·4 |
| *7* | Ro 07-1902 | II | $CH_2CH(OH)CH_2OCH_2CH{=}CH_2$ | — | 227 | −391[b] | 2·5[b] | 2·5 | 3·1, 2·4, 4·1, 3·3 |
| *8* | Ro 07-1502 | II | $CH_2CH(OH)CH_2OCH(CH_3)_2$ | — | 229 | −390[b] | 3·2[b] | 2·5 | 1·0, 1·0 |
| *9* | Benznidazole | II | $CH_2CONHCH_2$-Phenyl | — | 260 | −380[b] | 8·5[b] | 2·5 | 4·7, 2·2, 2·1 |
| *10* | Ro 31-0602 | II | $CH_2CH(OH)CH_2O\ (CH_2)_3CH_3$ | — | 243 | −388[c] | 13[c], 21[d] | 2·5 | 1·2, 0·4 |
| *11* | Ro 07-1127 | II | $CH_2CH(OH)CH_2O$Phenyl | — | 263 | −390[c] | 27[d], 31[c] | 2·5 | 0·0 |
| *12* | Ro 31-0752 | II | $CH_2CH(OH)CH_2O(CH_2)_{15}CH_3$ | — | 412 | −389[c] | >200[c] | 2·5 | 0·0 |
| *13* | Ro 03-8799 | II | $CH_2CH(OH)CH_2$-Piperidino-HCl | — | 290 | −346[c] | 8·5[f] | 2·5 | 0·7, 0·6 |
| *14* | RSU 1047 | II | $CH_2CH(OH)CH_2CH_2$-Morpholino | — | 270 | −375[g] | 0·34[h] | 2·5 | 0·8, 0·6 |
| *15* | Metronidazole | III | $CH_2CH_2OH$ | $CH_3$ | 171 | −486[b] | 0·96[b] | 2·5 | 1·0, 0·9 |
| *16* | Nimorazole | III | Morpholino | H | 226 | −457[b] | 1·4[b] | 2·5 | 1·4, 2·0 |
| *17* | NSC 38087 | IV | $CH_3$ | $SO_2O$ phenyl | 267 | −342[i] | 12[k] | 0·19 | 0·6, 0·0 |
| *18* | Azathioprine | IV | $CH_3$ | Thiopurine | 277 | −490[k] | 1·2[l] | 1·4 | 1·7, 1·5 |
| *19* | Nitrofurazone | V | $CH{=}NNHCONH_2$ | — | 198 | −257[b] | 1·7[b] | 0·63 | 0·8, 0·7 |
| *20* | Nitrofurantoin | V | $CH{=}NNCONHCOCH_2$ | — | 238 | −264[c] | 0·64[c] | 0·21 | 0·0, 0·2 |
| *21* | CB 1954 | VI | — | — | 252 | −385[m] | 1·4[m], 1·6[n] | 0·20 | 0·0, 0·0 |
| *22* | Duroquinone | VII | — | — | 164 | −244[i] | | 0·66 | 0·2, 0·1 |
| *23* | Menadione | VIII | — | — | 172 | −203[i] | 158[c] | 0·19 | 0·0, 0·4 |
| *24* | 9, 10-Anthraquinone 2-sulphonate, sodium | IX | — | — | 310 | −375[i] | <0·01[o] | 2·5 | 1·3, 0·4 |
| *25* | Imidazole | X | — | — | 68 | — | 0·83[p,c] | 2·5 | 0·0, 0·0 |
| *26* | SKF 525A | XI | — | — | 390 | — | | 0·13 | 4·7, 0·8, 3·3, 1·5 |

* See Fig. 1.

† One electron reduction potential (pH 7, 25°C) by pulse radiolysis or calculation.

‡ Partition coefficient for octanol/aqueous buffer (pH 7·4 or > $pK_1+2$ for *13* and *14*, 25 or 37°C), measured or calculated.

[a]See Brown & Workman (1980); [b]Adams *et al.* (1979); [c]Wardman, P. & Clarke, E. D. (personal communication); [d]Smithen, C. E. (personal communication); [e]Smithen *et al.*, (1980); [f]$pK_1=8·71$ (Smithen *et al.*, 1980), fraction un-ionized at pH 7·4 = 0·47, calculated apparent PC at pH 7·4 = 0·40; [g]Adams *et al.* (1980*b*); [h]$pK_1=6·65$ (O'Neill, P. & Hoe, S., personal communication), fraction un-ionized at pH 7·4 = 0·85, calculated apparent PC at pH 7·4 = 0·29; [i]Adams *et al.* (1980*c*) [j]Adams *et al.*, (1980*b*); [k]O'Neill, P. & Hoe, S. (personal communication); [l]O'Mahony, B. J. (personal communication); [m]Stratford *et al.* (1981); [n]Workman, P. (unpublished); [o]Fully ionized at pH 7·4, estimated apparent PC; [p]$pK_1=6·9$, fraction un-ionized at pH 7·4 = 0·75, calculated apparent PC at pH 7·4 = 0·62.

with HCl before injecting 0·04 ml/g. BENZO Ro 07-1127, nitrofurazone, nitrofurantoin, NSC 38087, azathioprine and anthraquinone sulphonate were suspended in 50% v/v polyethylene glycol (mol. wt 400, Sigma) in Hanks' and injected in 0·01 ml/g. Ro 31-0752, duroquinone and menadione were dissolved in arachis oil BP (McArthy's) and injected in 0·01 ml/g. CCNU was stored at −70°C and prepared within minutes of use. It was first dissolved in ethanol and then diluted 1:10 with 0·5% w/v carboxymethyl cellulose (BDH)/Hanks', immediately before i.p. injection in 0·01–0·02 ml/g.

Control experiments showed that none of the drug vehicles on their own affected tumour growth or response to CCNU. In screening experiments the following groups were always included: appropriate vehicle controls; CCNU 10 mg/kg alone; CCNU 10 mg/kg + test compound; CCNU 10 mg/kg + MISO 2·5 mmol/kg; test compound alone. In addition, CCNU 20 mg/kg was included in most experiments. Compounds were tested at 2·5 mmol/kg, but, if this proved toxic, doses close to maximum tolerated (MTD) were used.

The use of MISO as a positive control in each experiment allowed normalization of data to minimize between-experiment variation. We used an Activity Index (AI) for enhancement of CCNU effect, defined as:

$$AI = \frac{(GD_{x+CCNU}) - (GD_{x\ \mathrm{alone}}) - (GD_{CCNU\ \mathrm{alone}})}{(GD_{MISO+CCNU}) - (GD_{MISO\ \mathrm{alone}}) - (GD_{CCNU\ \mathrm{alone}})} \quad (1)$$

where $x$ is the test compound, GD is growth delay, the MISO dose is 2·5 mmol/kg and CCNU dose 10 mg/kg. In practice, there was no significant growth delay for MISO alone or for most test compounds; thus these terms were usually omitted. Using AI values, compounds were ranked for chemosensitization effectiveness against MISO (AI = 1).

Multiple regression analysis was carried out using the GLIM computer program (Version 3).

*Body temperature.*—At the doses used in this study, certain test compounds reduced mouse body temperature. In some experiments normal body termperatures were maintained in an incubator or with a lamp. Temperatures were measured with a rectal thermister probe and an electrical thermometer.

### RESULTS

### *Enhancement of CCNU response by MISO*

Fig. 2 shows typical data for the effect of MISO on the GD response of the KHT tumour to CCNU. The main effect is to remove the shoulder from the dose/response curve. The degree of enhancement is about the same for 2·5 as for 5 mmol/kg, and is similar whether MISO is given 30 min or immediately before CCNU. This is in good agreement with the GD and cell-survival data of Siemann (1981, 1982).

### *Enhancement of CCNU response by other agents*

*General.*—On the basis of the above experiments, a standard procedure was adopted for screening (see Methods). Compounds were given at 2·5 mmol/kg (or at MTD, if this was less) 30 min before 10 mg/kg CCNU. Except for CB 1954 and azathioprine, which gave barely significant but reproducible growth delays of about 1 day, none of the test compounds delayed growth when given alone.

The response to 10 and 20 mg/kg CCNU and 10 mg/kg CCNU plus 2·5 mmol/kg MISO were generally similar

FIG. 2.—Effect of MISO on the response of the KHT tumour to CCNU. ○, CCNU alone; □, 5 mmol/kg MISO and △, 2.5 mmol/kg immediately before CCNU; ■, 5 mmol/kg 30 min before CCNU.

FIG. 3.—Effect of partition coefficient on the enhancement of KHT tumour response to 10 mg/kg CCNU by neutral 2-nitroimidazoles ○, and 5-nitroimidazoles●, at 2.5 mmol/kg. For identification of analogues see Table. The line is the best fit for the quadratic relationship (see equation (3)). Points are means of activity indices in Table.

to those seen in Fig. 2. Data from 11 independent experiments gave the following median growth delays: 10 mg/kg CCNU, 1·5 days; MISO + 10 mg/kg CCNU, 4·5 days; 20 mg/kg CCNU, 10 days.

The abilities of the 26 test compounds to modify CCNU response are summarized in the Table. The results are expressed in terms of an Activity Index (AI; see Methods) which has a value of 1·0 for MISO. Compounds with AI values > 1·0 were more active than MISO, those with values < 1·0 were less active than MISO and those with zero values were totally inactive. Where supplies permitted, compounds were usually tested at least twice and the results were generally reproducible. A wide range of effectiveness is apparent, and it is important to identify the structural features responsible for activity.

*Neutral 2- and 5-nitroimidazoles.*—Consider first the activities of the 2- and 5-nitroimidazoles which are not charged at physiological pH. The neutral 2-nitroimidazoles are compounds *1–12* and the neutral 5-nitroimidazoles compounds *15* and *16* (Table). The PC values for these compounds vary over 4 orders of magnitude (0·016– > 200) with MISO intermediate (PC = 0·43). Fig. 3 shows the relationship between AI and log PC, and the curve is clearly bell-shaped, indicating a parabolic dependence. Analogues more hydrophilic than MISO (*i.e.* with lower PC) were inactive (the SR compounds *1–3*), or considerably less active than MISO (DEMIS, *4*). In contrast, those with PC 1–20 (*i.e.* more lipophilic) were usually more active, or at least as active, as MISO. The two most active Ro 07-1902 (*7*), and BENZO (*9*) have PC of 2·5 and 8·5 respectively. In many experiments these analogues increased the growth delay for 10 mg/kg CCNU to greater than that for 20 mg/kg CCNU alone, giving dose-modifying factors (DMF) in excess of 2. On the other hand, compound *8* with a similar PC (3·2) was no more active than MISO. The two most lipophilic analogues *11* and *12* were completely inactive.

The 2-nitroimidazoles all have electron affinities very similar to MISO ($E^1_7 \sim -390$ mV). The 5-nitroimidazoles metronidazole (*15*) and nimorazole (*16*) are considerably less electron-affinic, yet had activities similar or even superior to MISO (Table).

*Multiple regression analysis* was used to analyse the data for the neutral 2- and 5-nitroimidazoles showing measurable enhancement at 2·5 mmol/kg (*i.e.* compounds *4–10*, *15* and *16*). The data were fitted to a structure/activity relationship of the form (Hansch, 1971):

$$\log AI = b_2 \log PC + b_3 (\log PC)^2 + K \quad (2)$$

where $b_2$ and $b_3$ are constants calculated from the regression, and the constant $K$ was fixed to force the data through the MISO datum point. The mean AI was used for each compound, with a weighting equal to the product of AI and the square root of the number of estimations. The best fit ($\pm$s.e.) obtained was:

$$\log AI = (0{\cdot}472 \pm 0{\cdot}124) \log PC - (0{\cdot}567 \pm 0{\cdot}184) \log PC^2 + 0{\cdot}250 \quad n = 8,\ R^2 = 0{\cdot}71 \quad (3)$$

where $n$ is the number of data sets analysed in the regression, and $R^2$ the multiple-correlation coefficient, which shows that 71% of the variance in the data is explained by Equation (3). Omitting the quadratic term, the best fit obtainable was:

$$\log AI = (0{\cdot}228 \pm 0{\cdot}142) \log PC + 0{\cdot}0825 \quad n = 8,\ R^2 = 0{\cdot}25 \quad (4)$$

Only 25% of the variance is explained by Equation (4), and the improvement by including the quadratic term is highly significant ($0{\cdot}01 < P < 0{\cdot}025$, $F$ distribution on 1, 5 d.f.).

To determine the effect of electron affinity, the term $b_1$ ($E^1_7$) was added to the right-hand side of Equation (2). There was only a slight improvement in explained variance (78%) and this was not significant ($P > 0{\cdot}25$, $F$ distribution on 1, 5 d.f.). Thus, the data are best described by the quadratic relationship given in Equation (3).

*Others*.—The 2-nitroimidazoles *13* and *14* have basic substituents which are ionized at physiological pH. Neither was more effective than MISO (Table).

The remaining nitroheterocyclic compounds, comprising the highly electron-affinic nitrofurans nitrofurazone (*19*) and nitrofurantoin (*20*), the 5-substituted 4-nitroimidazoles NSC 38087 (*17*) and azathioprine (*18*), and the dinitrophenyl-azirdine CB 1954 (*21*) were toxic at 2·5 mmol/kg. At close to MTD nitrofurazone was reasonably active, but less than 2·5 mmol/kg MISO, whereas azathioprine was more active than MISO. The others had little or no activity.

Of the non-nitro electron-affinic agents. the two highly electron-affinic quinones, duroquinone (*22*) and menadione (*23*) were quite toxic, with little or no activity. The less electron-affinic anthraquinone sulphonate (*24*) was non-toxic at 2·5 mmol/kg and exhibited reasonable activity.

Two non-electron-affinic compounds were investigated. Imidazole (*25*) was ineffective at 2·5 mmol/kg and also at 5 mmol/kg (not shown). The hepatic microsomal-enzyme inhibitor SKF 525A (*26*) was active at 0·13 mmol/kg.

*Dose/response curves*.—The effect of the dose of the electron-affinic agent on the response of the KHT tumour to 10 mg/kg CCNU was determined for MISO (*5*), METRO (*15*) and the two most effective agents Ro 07-1902 (*7*), and BENZO (*9*). Fig. 4 shows combined data from a series of independent experiments. In these studies METRO was rather less active than MISO, whereas the others were confirmed as more active, even at low doses. Although Ro 07-1902 (*7*) was the most active at high doses, BENZO (*9*) showed good activity at doses down to 0·05–0·1 mmol/kg. The dose/response curve for BENZO is rather flat from 0·1 to 4 mmol/kg.

### *Effect of body temperature on response*

High doses of MISO, METRO and other lipophilic nitroimidazoles cause mice to become torpid and hypothermic (Workman & Brown, 1981). At 2·5 mmol/kg, MISO decreased body temperature by only 1–2°C and the hydrophilic analogues had no effect. Those more lipophilic than MISO produced a bigger decrease (up to 5–6°C) as did anthraquinone

FIG. 4.—Dose/response curves for the enhancement of the response of the KHT tumour to 10 mg/kg CCNU by various nitroimidazoles. The responses to 10 and 20 mg/kg CCNU without the addition of nitroimidazoles are also shown (◇, ◆). The data were obtained in two independent experiments, indicated by the open and closed symbols.

sulphonate (*24*). Ro 31-0602 (*10*) at 2·5 mmol/kg appeared to cause temporary paralysis, but this was not seen at lower doses.

It seems unlikely that these effects make a major contribution to tumour response. Very high doses of METRO (*e.g.* 10 mmol/kg) reduced temperature by ~10°C but the tumour response was about the same as with 2·5 mmol/kg MISO (Fig. 4). Furthermore, by reducing the dose of the lipophilic analogues, good tumour responses were maintained (Fig. 4) without a marked temperature fall. In three experiments with the active lipophilic analogue Ro 07-1902 (*7*) tumour response to 10 mg/kg CCNU plus 2·5 mmol/kg Ro 07-1902 was determined in mice whose body temperatures either were allowed to fall to about 32°C or were maintained at 36–37°C in an incubator or with a lamp. There was no difference in tumour response.

DISCUSSION

These studies, along with several others (Siemann, 1981, 1982; Twentyman, 1981; Hirst *et al.*, 1982) demonstrate that the response of the KHT tumour to CCNU can be increased considerably by MISO. Enhancement of *in vivo* tumour response to CCNU by 2·5–5 mmol/kg MISO has been demonstrated also with the RIF-1 sarcoma (Siemann, 1981, 1982; Twentyman, 1981; Twentyman & Workman, 1982), Lewis lung carcinoma (Stephens *et al.*, 1981), MT-1 mammary tumour (Siemann, 1982), SCC VII/St carcinoma and EMT6/St/1u tumour (Hirst *et al.*, 1982). We are not aware of any experiments where these doses of MISO have failed to enhance response to CCNU. In addition, doses of 2·5–5 mmol/kg MISO enhanced tumour response to methyl-CCNU and BCNU in most tumours evaluated (Clement *et al.*, 1980; Stephens *et al.*, 1981; Tannock, 1980*b*; Mulcahy *et al.*, 1981; Clutterbuck *et al.*, 1982), and MISO also enhanced KHT tumour response to PCNU but not to chlorozotocin (Mulcahy, 1982). Taken overall, chemosensitization with CCNU appears to be at least as good as that seen for other cytotoxics.

To find the best sensitizer-nitrosourea combinations, detailed structure/activity studies are required. The present paper gives data for 26 compounds, mostly electron-affinic, in combination with CCNU against the KHT tumour. By analogy with structure/activity relationships for radiosensitization (*e.g.* Adams *et al.*, 1979; Anderson *et al.*, 1981) we were particularly interested in the effects of lipophilicity and electron-affinity.

For the neutral 2- and 5-nitroimidazoles, PC varied from 0·014– > 200. Of those more hydrophilic than MISO the 3 SR compounds (*1–3*) were completely inactive at 2·5 mmol/kg, and DEMIS (*4*) considerably less effective. Siemann (1982) observed similar results with SR-2508 (*3*), SR-2555 (*2*) and DEMIS at 5 mmol/kg. Some enhancement of RIF-1 tumour response was obtained with SR-2508, but only at high CCNU doses ( > 20 mg/kg) and less than obtained with MISO. A similar lack of effect of SR-2508 was also seen by Hirst *et al.* (1982).

We observed much greater activity with lipophilic nitroimidazoles. At 2·5 mmol/kg, several MISO analogues with PC 1–10 gave superior enhancement of CCNU response (Fig. 3). Furthermore, Ro 07-1902 (*7*) and particularly BENZO (*9*) were active at much lower doses than MISO (Fig. 4). The superior activity of BENZO has been confirmed recently by Siemann (personal communication). Lipophilicity cannot be increased much further, however, before activity is lost, the overall dependence being parabolic (Fig. 3).

It was not possible to examine the influence of electron affinity on enhancement of CCNU response in quite as much depth, because of the toxicity of the highly electron-affinic compounds. Nevertheless, enhancement data were obtained for compounds with $E^1_7$ values from −486 to −203 mV, and are sufficient to demonstrate that electron affinity is less important than lipophilicity. For the neutral 2- and 5-nitroimidazoles, multiple regression analysis revealed no significant advantage in including an electron-affinity term into the quadratic expression for lipophilicity. Of the compounds more electron-affinic than MISO, only nitrofurazone (*19*) showed comparable enhancement of CCNU response at MTD (Table). The other 5-nitrofuran, nitrofurantoin (*20*), was virtually inactive, as were the non-nitro compounds duroquinone (*22*) and menadione (*23*). The two quinones are very lipophilic, which may contribute to their lack of enhancement; however, the nitrofurans have PC values close to the optimal range for nitroimidazoles. Another non-nitro compound anthraquinone-2-sulphonate (*24*) has similar electron affinity to MISO, was less toxic than the more electron-affinic quinones, and showed reasonable enhancement at 2·5 mmol/kg, despite its hydrophilicity.

It is useful to compare our structure/activity relationships for enhancement of CCNU response *in vivo* with those for radiosensitization. *In vitro* radiosensitization of hypoxic cells by neutral MISO analogues is largely dependent on electron affinity. Although some effect is seen at extreme PC values (Anderson *et al.*, 1981; Brown *et al.*, 1982) lipophilicity has relatively little influence. *In vivo* structure/activity relationships are inevitably more complex, because of pharmacokinetic considerations, and this applies to both radiosensitization and chemosensitization. However, selected examples will demonstrate that the structural features required for the two effects are rather different. For example, Rauth *et al.* (1978) evaluated the 2-nitroimidazoles *4*, *5*, *6*, *7* and *11* for *in vivo* radiosensitization, using the same tumour and mouse strain and similar timing to the present study. Ro 07-1902 (*7*) showed radiosensitization comparable to MISO, whereas it is considerably more active in enhancing the CCNU response. Both the hydrophilic DEMIS (*4*) and the lipophilic Ro 07-1127 (*11*) gave good radiosensitization, but have little or no ability to enhance the CCNU response. Pharmacokinetic considerations are clearly not responsible for the inactivity of the hydrophilic analogues DEMIS, SR-2508 and SR-2555 with CCNU, since these achieve plasma and tumour levels sufficient for radiosensitization comparable to MISO (Brown & Workman, 1980; Brown *et al.*, 1981). They may, however, contribute to the inactivity of the most lipophilic and the most electron-affinic analogues. Quantitative differences in the pharmacokinetics of MISO between experimental animals and man are well established (Workman & Brown, 1981) and therefore caution should be exercised in extrapolating chemosensitization data directly to man. Pharmacokinetic studies are in progress with this series and some preliminary data have been reported (White *et al.*, 1982). It should be noted that the ability of the markedly hydrophilic anthraquinone-2-sulphonate (*24*) to enhance CCNU response may be due to free anthraquinone, since sulphonate groups are rapidly metabolized in mice (Zanelli & Kaelin, 1981).

Recent *in vitro* studies have identified nitroimidazoles which radiosensitize hypoxic mammalian cells more efficiently than predicted from their electron affinities These include 2-nitroimidazoles with basic alkanolamine substituents partially protonated at physiological pH, and 5-substituted 4-nitroimidazoles (Adams *et al.*, 1980*a*; Smithen *et al.*, 1980). We tested two alkanolamines, Ro 03-8799 (*13*) and RSU 1047 (*14*). At 2·5 mmol/kg both enhanced CCNU activity slightly less than MISO (Table). Two 5-substituted 4-nitroimidazoles were examined, NSC 38087 (*17*) and azathioprine (*18*). The former was highly toxic and had little activity at MTD. In contrast, azathioprine at 1·4 mmol/kg was superior to 2·5 mmol/kg MISO (Table). The dinitrophenylaziridine CB 1954 (*21*) is also a better-than-predicted radiosensitizer of hypoxic mammalian cells (Stratford *et al.*, 1981, but was rather toxic and gave no enhancement of CCNU response at MTD (Table).

The mechanism of enhancement of nitrosourea response is unknown, but the main possibilities have been discussed (Siemann, 1981, 1982; Mulcahy *et al.*, 1981; Brown, 1982). Enhancement of CCNU response was seen with EMT6 tumour spheroids after prolonged hypoxic pre-exposure to MISO *in vitro*, and was not due to inhibition of recovery from potentially lethal damage (Twentyman, 1982). In contrast, inhibition of clonogenic cell recovery was seen with the KHT tumour *in vitro* and carbamoylation of repair enzymes may be involved (Mulcahy, 1982). Depletion of glutathione by MISO has been demonstrated (Brown, 1982). Tannock (1980) showed that serum from mice receiving MISO and BCNU (but not BCNU alone) was preferentially cytotoxic to hypoxic cells. Our studies show that enhancement of CCNU response is not due to reduced body temperature, and this has been confirmed by Siemann (personal communication).

Structure/activity relationships present another approach to the mechanism. The present studies show that the structural features responsible for the enhancement of the KHT tumour response are different from those affecting radiosensitization; lipophilicity being more important and electron affinity less so. Despite having a PC slightly greater than MISO, imidazole (*25*) was inactive. This may indicate a requirement for some electron affinity, though not necessarily in a nitro group, since anthraquinone sulphonate (*24*) was also effective. On the other hand we demonstrated that, despite having no electron affinity, SKF 525A (*26*) gave good enhancement of CCNU response, a result confirmed recently by Siemann (personal communication). SKF 525A also increased the response of the ascites L1210 leukaemia to methyl-CCNU in mice, as well as reducing the $LD_{50}$ (Klubes *et al.*, 1979). A possible mechanism which might be shared by SKF 525A and the nitroimidazoles of intermediate lipophilicity would be one operating through inhibition of re-routing of nitrosourea metabolism. This could accommodate (1) the absence of enhancement of CCNU response by hydrophilic analogues which are not metabolized, but cleared by the kidney (Workman & Brown, 1981) and thus are unlikely enzyme inhibitors, (2) the lack of enhancement of response to the hydrophilic nitrosourea chlorozotocin, also cleared by the kidney (Hoth *et al.*, 1978) and (3) the lack of enhancement of CCNU response by the highly electronaffinic and other toxic analogues, because of inadequate concentrations for inhibition.

The principal aim of this investigation was to identify for further attention compounds which might be superior chemosensitizers to MISO. The lipophilic analogues, particularly Ro 07-1902 and BENZO, clearly warrant more detailed evaluation. BENZO is of particular interest because the tumour enhancements reported here occur with doses much lower than with MISO, and plasma and tumour concentrations corresponding to active doses in mice can be maintained for several hours in dogs (White *et al.*, 1982). Daily doses of up to 0·03 mmol/kg BENZO have been used in the treatment of Chaga's disease in man (Coura *et al.*, 1978) and the drug is eliminated with a half-life similar to MISO (Raaflub, 1980). Preliminary studies in mice have demonstrated a therapeutic gain for combining BENZO with CCNU, and BENZO is now undergoing preliminary clinical evaluation as a chemosensitizer in this Unit.

In view of the therapeutic advantage which has been reported for the combination of MISO with CCNU (Siemann, 1981, 1982; Twentyman & Workman, 1982*a*; Hirst *et al.*, 1982) we are now evaluating the effect of combining BENZO or Ro 07-1902 with CCNU, and also with other cytotoxics for which they show improved enhancement over MISO, *i.e.* chlorambucil and cyclophosphamide (Twentyman & Workman, 1982*b*) and Melphalan (Sheldon & Batten, 1982; Clutterbuck & Miller, personal communication). We are also optimizing the dose schedules, with emphasis on relevance to clinical pharmacokinetics.

We thank Dr C. E. Smithen of Roche (Welwyn) for supplies of the Roche analogues; Dr J. M. Brown, Department of Radiology, Stanford University, and Dr W. W. Lee of SRI for the SR compounds; Dr I. J. Stratford, Institute of Cancer Research, Sutton, for NSC 38087 and RSU 1047; Dr D. E. V. Willman, Chester Beatty Research Institute, London, for CB 1954; May and Baker for metronidazole; Farmitalia Carlo Erba for nimorazole; Dr V. L. Narayan, Drug Synthesis and Chemistry Branch, NCI, and Lundbeck for CCNU. We are grateful to Drs Smithen, P. Wardman (Gray Laboratory), Stratford and P. O'Neil (ICR) for unpublished physicochemical data, and to Mr L. S. Freedman and Petra Macaskill (MRC, Cambridge) for the multiple linear regression analysis. We also thank Jane Donaldson, Daryl Knight, Nancy Smith, Jill Shaw and Kate Smith for excellent technical assistance.

### REFERENCES

Adams, G. E., Ahmed, I., Clarke, E. D. & 6 others (1980*a*) Structure–activity relationships in the development of hypoxic cell radiosensitizers. III. Effects of basic substituents in nitroimidazole side-chains. *Int. J. Radiat. Biol.*, **38**, 613.

Adams, G. E., Ahmed, I., Fielden, E. M., O'Neill, P. & Stratford, I. J. (1980*b*) The development of some nitroimidazoles as hypoxic cell sensitizers. In *Radiation Sensitizers* (Ed. Brady). New York: Masson, p. 33.

Adams, G. E., Clarke, E. D., Flockhart, I. R. & 8 others (1979) Structure–activity relationships in the development of hypoxic cell radiosensitizers. I. Sensitization efficiency. *Int. J. Radiat. Biol.*, **35**, 133.

Adams, G. E., Flockhart, I. R., Smithen, C. E., Stratford, I. J., Wardman, P. & Watts, M. E. (1976) Electron-affinic sensitization VII. A correlation between structures, one-electron potentials and efficiencies of nitroimidazoles as hypoxic cell radiosensitizers. *Radiat. Res.*, **67**, 9.

Adams, G. E. Stratford, I. J., Wallace, R. G., Wardman, P. & Watts, M. E. (1980*c*) Toxicity of nitro compounds towards hypoxic mammalian cells *in vitro*: Dependence on reduction potential. *J. Nat. Cancer Inst.*, **64**, 555.

Anderson, R. F., Patel, K. B. & Sehmi, D. S. (1981) Radiosensitization of hypoxic bacterial cells by nitroimidazoles of low lipophilicity: Steady state and rapid mix studies. *Radiat. Res.* **85**, 496.

Brown, J. M. (1982) The mechanism of cytotoxicity and chemosensitization by misonidazole and other nitroimidazoles. *Int. J. Radiat. Oncol. Biol. Phys.*, **8**, 675.

Brown, J. M., Brown, D. M. & Lee, W. W. (1981) SR-2508: A 2-nitroimidazole amide which should be superior to misonidazole as a radiosensitizer for clinical use. *Int. J. Radiat. Oncol. Biol. Phys.*, **7**, 695.

Brown, D. M., Parker, E. T. & Brown, J. M. (1982) Structure–activity relationships of 1-substituted-2-nitroimidazoles: Effect of partition coefficient and side-chain hydroxyl groups on radiosensitiation *in vitro*. *Radiat. Res.*, **90**, 98.

Brown, J. M. & Workman, P. (1980) Partition coefficient as a guide to the development of radiosensitizers which are less toxic than misonidazole. *Radiat. Res.*, **82**, 171.

Clutterbuck, R. D., Millar, J. L. & McElwain, T. J. (1982) Misonidazole enhances the action of BCNU and melphalan against human melanoma xenografts. *Am. J. Clin. Oncol.*, **5**, 73.

Coura, J. R., Brindeiro, P. J. & Ferreira, I. (1978) Benznidazole in the treatment of Chaga's disease. In *Current Chemotherapy* (Eds Siegenthaler & Luthy). Washington: American Society for Microbiology. p. 161.

Hansch, C. (1971) Quantitative structure–activity relationships in drug design. In *Drug Design*,

Vol 1 (Ed. Ariens). New York: Academic Press. p. 271.

HIRST, D. G., BROWN, J. M. & HAZELHURST, J. (1982) Enhancement of CCNU cytotoxicity by misonidazole: Studies of possible therapeutic gain. *Br. J. Cancer*, **46**, 109

HOTH, D., WOOLLEY, P., GREEN, D., MACDONALD, J. & SCHEIN, P. (1978) Phase 1 studies on chlorozotocin. *Clin. Pharmacol. Ther.*, **23**, 713.

KALLMAN, R. F., SILINI, V. & VAN PUTTEN, L. M. (1967) Factors influencing the quantitative estimation of the *in vivo* survival of cells from solid tumors. *J. Natl Cancer Inst.*, **39**, 539.

KLUBES, D., MILLAR, H. G., CERNA, I. & REVETHICK, J. (1979) Alterations in the toxicity and antitumor activity of methyl-CCNU in mice following pretreatment with either phenobarbitone or SKF 525A. *Cancer Treat. Rep.*, **63**, 1901.

MCNALLY, N. J. (1982) Enhancement of chemotherapy agents. *Int. J. Radiat. Oncol. Biol. Phys.*, **8**, 593.

MULCAHY, R. T. (1982) Chemical properties of nitrosoureas: Implications for interaction with misonidazole. *Int. J. Radiat. Oncol. Biol. Phys.* **8**, 599.

MULCAHY, R. T., SIEMANN, D. W. & SUTHERLAND, R. M. (1981) *In vivo* response of KHT sarcoma to combination chemotherapy with radiosensitizers and BCNU. *Br. J. Cancer*, **43**, 93.

RAAFLUB, J. (1980) Multiple-dose kinetics of trypanosomicide benznidazole in man. *Arzneim. Forsch.*, **30**, 2192.

RAUTH, A. M., CHIN, J., MARCHOW, L. & PACIGA, J. (1978) Testing of hypoxic cell radiosensitizers *in vivo*. *Br. J. Cancer*, **37**, (Suppl. III), 206.

SHELDON, P. W. & BATTEN, E. L. (1982) Potentiation *in vivo* of melphalan activity by nitroimidazole. compounds *Int. J. Radiat. Oncol. Biol. Phys.*, **8**, 635.

SIEMANN, D. W. (1981) *In vivo* combination of misonidazole and the chemotherapeutic agent. CCNU *Br. J. Cancer*, **43**, 367.

SIEMANN, D. J. (1982) Response of murine tumours to combinations of CCNU with misonidazole and other radiation sensitizers. *Br. J. Cancer*, **45**, 272.

SMITHEN, C. E., CLARKE, E. D., DALE, J. A. & 4 others (1980) Novel (nitro-1-imidazolyl)-alkanolamines as potential radiosensitizers with improved therapeutic properties. In *Radiation Sensitizers* (Ed. Brady). New York: Masson. p.22.

STEPHENS, T. C., COURTENAY, V. D., MILLS, J., PEACOCK, J. H., ROSE, C. M. & SPOONER, D. (1981) Enhanced cell killing in Lewis lung carcinoma and human pancreatic-carcinoma xenograft by the combination of cytotoxic drugs and misonidazole. *Br. J. Cancer*, **43**, 451.

STRATFORD, I. J., WILLIAMSON, C., HOE, S. & ADAMS, G. E. (1982) Radiosensitizing and cytotoxicity studies with CB 1954. *Radiat. Res.*, **88**, 502.

TANNOCK, I. F. (1980) *In vivo* interaction of anticancer drugs with misonidazole or metronidazole: Cyclophosphamide and BCNU. *Br. J. Cancer*, **42**, 871.

TWENTYMAN, P. R. (1981) Modification of tumour and host response to chemotherapy by misonidazole or by WR 2721. *Br. J. Radiol.*, **54**, 369.

TWENTYMAN, P. R. (1982) *In vitro* preincubation with misonidazole under hypoxic conditions: Effect on drug response of EMT6 spheroids. *Int. J. Radiat. Oncol. Biol. Phys.*, **8**, 607.

TWENTYMAN, P. R., KALLMAN, R. F. & BROWN, J. M. (1979) The effect of time between X-irradiation and chemotherapy on the growth of three solid mouse tumours. 1. Adriamycin. *Int. J. Radiat. Oncol. Biol. Phys.*, **5**, 1255.

TWENTYMAN, P. R. & WORKMAN, P. (1982*a*) The effect of misonidazole or metronidazole pretreatment on the response of the RIF-1 mouse sarcoma to melphalan, cyclophosphamide, chlorambucil and CCNU. *Br. J. Cancer*, **45**, 447.

TWENTYMAN, P. R. & WORKMAN, P. (1982*b*) The effect of radiosensitizer pretreatment on the response of the RIF-1 mouse sarcoma to cytotoxic drugs. *Int. J. Radiat. Oncol. Biol. Phys.* (In press.)

WHITE, R. A. S., WORKMAN, P. & OWEN, L. N. (1982) The pharmacokinetics in mice and dogs of nitroimidazole radiosensitizers and chemosensitizers more lipophilic than misonidazole. *Int. J. Radiat. Oncol. Biol. Phys.*, **8**, 473.

WORKMAN, P. (1980) Pharmacokinetics of hypoxic cell radiosensitizers. A review. In *Radiation Sensitizers* (Ed. Brady). New York: Masson. p. 192.

WORKMAN, P. & BROWN, J. M. (1981) Structure–pharmacokinetic relationships for misonidazole analogues in mice. *Cancer Chemother. Pharmacol.* **61**, 39.

WORKMAN, P. & TWENTYMAN, P. R. (1981) Structure/activity relationships for the enhancement of the anti-tumour effect of CCNU by electron affinic agents. *Br. J. Cancer*, **44**, 283.

WORKMAN, P. & TWENTYMAN, P. R. (1982) Enhancement by electron-affinic agents of the therapeutic effects of cytotoxic agents against the KHT tumour: structure/activity relationships. *Int. J. Radiat. Oncol. Biol. Phys.*, **8**, 623.

ZANELLI, G. D. & KAELIN, A. Z. (1981) Synthetic porphyrins as tumour-localizing agents. *Br. J. Radiol.*, **54**, 403.

*Note added in proof:* We have recently examined sensitization of the KHT tumour to CCNU by 1-(2-hydroxy-3-methoxypropyl) - 2 - methyl - 4 - nitroimidazole (Watras *et al.*, 1979, *Br. J. Cancer*, **40**, 354). This is slightly more lipophilic (PC = 0·96) than MISO, but considerably less electron-affinic ($E^1_7 = -564$ mV) than any of the nitroimidazoles reported here. At 2·5mmoles/kg enhancement (AI = 1·3) was superior to MISO, thus confirming the predominance of lipophilicity over electron affinity in this system.